# Using student feedback about the learning environment as a starting point for co-construction

**DOI:** 10.1007/s10984-021-09403-9

**Published:** 2022-01-07

**Authors:** Jill M. Aldridge, Silvana Bianchet

**Affiliations:** 1 School of education, Curtin University, GPO Box U1987, 6845 Perth, WA Australia; 2 Aloysius College, 53 Wakefield Street, 5000 Adelaide, South Australia Australia

## Abstract

The context in which learning takes place, or learning environment, is pivotal to a positive learning experience for students. Although numerous studies have established strong links between a positive learning environment and a range of student outcomes, far less research has examined how teachers might establish such an environment. Amidst growing acknowledgment that opportunities for the co-construction of learning and assessment design could provide a means of developing a more positive learning environment, this case study examined one such journey. Using a case study approach, we argue that student feedback involving a learning environment survey provides a valuable starting point for including students in co-construction and classroom improvement. Our findings indicate that teachers can improve the learning environment by involving students in meaningful co-construction through open tasks.

## Introduction

This study took place amidst a reform effort in Catholic schools in South Australia (CESA). The new Living, Learning, Leading framework, introduced to guide the reform efforts, requires the implementation of a co-constructed curriculum and learning and assessment design. Co-constructed curriculum and learning and assessment design (or co-construction) involves redefining the nature of learning and relations between teachers and students (from vertical relationships fostering compliance to horizontal relationships involving mutual influence and dialogue) (Rincón-Gallardo & Elmore, 2012). Co-construction requires student empowerment and agency, making the process consistent with critical pedagogy (Freire) and progressive education (Dewey, Montessori).

This article examines the importance of developing a positive learning environment and why this could be central to school and classroom improvement. It then examines co-construction and its use in past school improvement efforts, including ‘student voice’. Finally, we report the results of a case study of how one teacher (second author) used the Classroom Climate Questionnaire (CCQ) to facilitate co-construction, leading to open tasks.

## Background

### The learning environment: A key component of the learning experience

The psychosocial learning environment, the context in which learning takes place, is influenced by a range of factors, including social, cultural, psychological influences and the types of learning activities and assessments provided by the teacher (Afari et al., 2013). The learning environment is, in effect, a system in which learning occurs, and it is the students who are at the heart of it. Given the length of time that students spend in classrooms, up to 15,000 h by the time they leave high school (Rutter, 1979), and the fact that they are major stakeholders in the education process, it is logical to suggest that their views are an essential consideration.

Learning environment research has provided consistent evidence to suggest that “students learn better when they view the classroom environment more positively” (see, for example, Dorman & Fraser, 2009, p. 78). A plethora of research has found strong and consistent links between students’ perceptions of their learning environment and a range of outcomes (see, for example, a review by Fraser, 2014). Given the overwhelming and consistent findings, it is reasonable to suggest that changing psychosocial aspects of the learning environment may influence students’ outcomes. There is, however, less research that investigates what teachers can do to change the learning environment in ways that better suit students’ learning. The field of learning environments has, over the past 50 years, developed a wide range of valid and reliable instruments that can help to do just that, tap into students’ perceptions of the learning environment (Fraser, 2018). Given that school improvement efforts strive for improved student outcomes, developing rich, dynamic learning environments that help students become better learners makes sense.

The students are at the centre of the learning environment, whose lives are interwoven with experiences that are coloured by friendships, identities, and expectations. These experiences and teachers’ reactions to the experience will impact the learning environment and how students experience it. Without an understanding of the lives and needs of the students, creating an effective learning environment becomes difficult. Co-construction provides a shared account as the basis for meaningful change in school improvement efforts.

Evidence suggests that teachers’ reflection on student feedback can lead to positive changes in students’ perceptions of the learning environment (Aldridge et al., 2012; Bell & Aldridge, 2014). Using a five-step process (see www.NSIpartnerships.com.au for a copy of the guide), with reflection at the heart of the process, teachers have successfully planned and implemented pedagogical change linked to school improvement (see, for example, Aldridge et al., 2021). While past research demonstrates that student feedback is valuable, the involvement of students beyond completing the survey is often limited to providing information related to why they responded to items in the survey the way they did. The research described in this article builds on this past research by examining how one teacher used co-construction, which involved student feedback to a learning environment survey as a starting point, to work meaningfully with students to bring about pedagogical changes, helping to fill this gap in the research.

### Co-Construction: Engaging students in the improvement process

Although the education process is for the students’ benefit, much of what occurs in schools and classrooms does not consider these stakeholders’ views. Many school improvement efforts have omitted student voices altogether (Lodge, 2005). However, a relatively recent trend has emerged to involve students in the improvement process. The pervading view concerning student involvement is that what students have to say “… about teaching, learning, and schooling, is not only worth listening to but provides an important—perhaps the most important—foundation for thinking about ways of improving schools” (Rudduck et al., 1996, p. 1).

Incorporating student voice can improve the overall culture or environment of the school (see, for example, Aldridge et al., 2021; Read et al., 2015) and student achievement (Friend & Caruthers, 2015). However, it is not without its challenges (Voight, 2015). There is a range of views about the extent to which students should be empowered to make changes (Lodge, 2005) which, in turn, leads to the contentious issue of the purpose of including students’ voice in school improvement. There are reports of limited and tokenistic student involvement (see, for example, Charteris & Smardon, 2019; Lodge, 2008; Yonezawa & Jones, 2007; Jones & Yonezawai 2002). Lodge, (2008) identified uses of student’s voice that are not helpful to school improvement, including: as quality control (e.g., through the use of satisfaction surveys or similar); as a source of information (that do not extend beyond the initial feedback); as a means of promoting compliance in students (and teachers), and control and surveillance (e.g., the opportunity for the student to be critical of teachers). These forms of student involvement serve to disempower students.

Examining ways to empower students and provide agency is essential for student voice to be helpful. Involving students should provide opportunities to listen to students and actively apply their views in meaningful ways. There have been some examples of successful means of using student voice, such as counter-public forums (Diera 2016); student interviews (Friends & Caruthers, 2012); participatory action research (O’Neill & McMahon, 2012); and students as co-researchers (Susino & Haya, 2014; Yonezawa & Jones, 2009). In each of these cases, student involvement has provided meaningful improvement.

Although learning environment research has provided a valuable means of allowing teachers to gather student feedback about the learning environment (Bell & Aldridge, 2014), research related to how students can be involved beyond responding to a survey is scarce. Our study filled this gap by examining how one teacher went further to use a classroom learning environment survey to provide students with meaningful input into the co-construction of the learning and assessment design and whether this input improved their perceptions of the learning environment. In this article, we argue that a process criterion approach (as opposed to a focus on student outcomes), involving a learning environment survey, could be an effective means of determining the efficacy of new or innovative teaching approaches.

### Context

In South Australia, Catholic schools are examining ways to implement co-construction as part of a system-wide reform effort. The notion of co-construction draws on Dewey’s (1916) progressive education theory, Piaget’s (1972) social constructivist theory, and Freire’s (1973) problem-solving model. Co-construction is a term that has been used in a variety of educational settings (see, for example, in language learning). However, at the heart of co-construction is Vygotsky’s (1962) notion of shared understanding or shared meaning of learning. In co-construction, students become active participants in their learning and, in this way, co-construction becomes a process in which students and the teacher “communicate, share the construction of knowledge and develop new understandings that create sustainable learning” (Lodge, 2005, p. 132). In school improvement, co-construction engages and empowers students to bring about a shared understanding of learning, leading to classroom and school change. Co-construction allows students the opportunity not only to present their viewpoint but also empowers them to be protagonists in their learning. In this way, students become agents of change, or ‘actors with alternatives’, rather than passive learners (Ertmer & Newby, 1996). If students are to be given a voice and a propensity to be active participants in their learning, then it makes sense to involve co-construction of the learning environment.

To date, the reform is in its infancy, with most teachers grappling to understand what co-construction means and what it might look like, and how to incorporate it into their classroom. Therefore, this study will benefit these teachers by providing information about one teachers’ journey and how her efforts impacted students’ perceptions of the learning environment.

## Methods

The study involved a case study of one teacher and her journey to co-construct the learning environment with students in her Italian classes. The study examines how she used student feedback about the learning environment (using the Classroom Climate Questionnaire, CCQ) to help her examine whether co-construction (using open tasks) improved students’ perceptions of the learning environment.

A qualitative case study was embraced, as it allowed us to explore the phenomenon under study within its real-life context (Yin, 2003) and collect rich and naturalistic data (Stake, 1995). The phenomenon under investigation was the teacher’s use of a learning environment survey and the subsequent implementation of ‘open tasks’ to engage students in co-construction and improve the learning environment. A case study was considered appropriate as it allowed us to focus on this teacher’s journey as she worked with students. The teacher was selected because she used the CCQ to improve her teaching practice for several years. Over this time, she developed and implemented interventions that promoted co-construction of the curriculum and learning design.

The study was carried out in an all-girls school in South Australia. The data was collected from students in two high school language (Italian) classes selected by the case study teacher. The two classes, a year 8 class and a year 10 class, were taught by the teacher (second author); the year 8 class comprised 25 students, and the year 10 class included 18 students.

### Data collection

Data were collected predominantly through three different methods: interviews, narratives, and survey data. Interviews are advantageous as they help describe and explain people’s actions and behaviours. As Yin (2003, p. 92) stated, “Interviews are an essential source of case study evidence since most case studies are about human affairs”. Discussion style interviews with students were carried out throughout the study by the teacher (second author). These interviews became part of the teaching and learning process and were used to reflect on and, if required, used to change the processes involved in the intervention. Interviews between the researcher (first author) and the teacher, each lasting one hour, involved a semi-structured format, allowing scope for a more conversational style and being responsive to a lead in the teacher’s direction while ensuring that the interviews remained focused on key issues.

Student-generated narratives were used to elicit students’ responses to the open-ended task and their role in co-construction. Although there are advantages to face-to-face interviews, the onset of COVID-19 precluded the opportunity for the first author to do this. Although other forms of gathering data were considered, narratives were thought to be the most suitable method as they provided rich information and were largely unobtrusive (compared to observations). As a human endeavour, Narration contributes to our understanding of ourselves and the world we live in (Wiklund-Gusin 2010). Using student narratives provided us with insights into students’ thinking and self-understanding. In our interpretation of the narratives, we were cognisant that, while the students achieved self-understanding, this was dependent upon the ‘regard, words and actions of others” (Wiklund-Gustin, 2010, p. 32). Thematic analysis was used to analyse the narratives.

A learning environment survey, the Classroom Climate Questionnaire (CCQ; see Aldridge et al., 2021), was used to assess students’ perceptions of the learning environment. The CCQ assesses eleven malleable features of the learning environment (referred to as scales). These scales categorised into three broad dimensions, which assess: the relationships within the class (including three scales; student cohesiveness, teacher support, and equity), the assessment practices used (including two scales; feedback and clarity of assessment criteria), and the delivery of lessons in terms of high impact teaching strategies (including six scales; responsibility for learning, involvement, personal relevance, collaboration, task orientation, and differentiation). Table 1 provides a short description of each scale along. (For information on the internal consistency reliability and why each scale is important to the learning environment, see Aldridge et al., 2021.) A valuable feature of the CCQ, is the ability for students to respond to each item twice using a side-by-side format. This feature allows students to indicate their perceptions of how often each statement occurs in the classroom (actual learning environment) and how often they would prefer the statement to occur (preferred learning environment). These responses provide teachers with information that supports critical self-reflection and guides student discussions. The individual statements or items are responded to using a five-point frequency format of almost never, rarely, sometimes, often, and almost always.

**Table 1:** Description and sample item for each CCQ scale

Scale		Description
RELATIONSHIPS		*Assesses the extent to which …*
	Student Cohesiveness	… there is an environment in which students feel accepted and supported by their classmates and safe to express their ideas.
	Teacher Support	… the teacher helps, cares about, trusts, and is interested in students.
	Equity	… students are treated fairly by the teacher.
	Responsibility for Learning	… teachers treat students with respect and give students responsibility for their learning.
ASSESSMENT	Clarity of Assessment	… the assessment requirements and criteria are explicit, and the basis for judgments is clear.
	Feedback	… students feel that the feedback provided by the teacher is timely, makes a positive contribution to their learning, and redirects or refocuses learning.
DELIVERY	Involvement	… students feel that there is scope to be involved in the lessons, including participating in discussions, asking questions, and sharing ideas.
	Task Orientation	… it is important to set goals, complete activities, and stay on the subject matter.
	Personal Relevance	… the subject is relevant to students’ every-day and out-of-school experiences.
	Collaboration	… students are engaged in collaborative learning experiences and work with one another on learning tasks.
	Differentiation	… teachers cater to students differently based on ability, rates of learning, and interests.

In our study, the CCQ was administered twice in each class, once as a pre-test and, eight weeks later, as a post-test. This data was used in two ways. First, pre-test data provided a point of reflection for the students and the teacher and as a springboard for decisions concerning how to improve the classroom learning environment (elaborated below). Second, the teacher compared the pre-test and post-test results to indicate whether and in what areas the teachers’ efforts successfully improved the learning environment.

To provide the teacher with information related to student responses to the CCQ, the teacher received a variety of data, including, but not limited to, the average item mean (calculated separately for each scale) and standard deviation.

Paired sample *t-*tests were used to examine whether the pre-post differences were statistically significant. On the other hand, we calculated the effect sizes (as recommended by Thompson, 2007) to examine the magnitude of the pre-post differences for the different learning environment scales. The effect sizes were calculated by subtracting the pre-test’s mean from the post-test’s mean and dividing the results by the pooled standard deviation from the groups sampled.

$$ {\rm{Cohen's}}\,d =\frac{{M_1}-{M_2}}{\it{SD}_{pooled} }\quad  $$

## Results

**Fig. 1:**
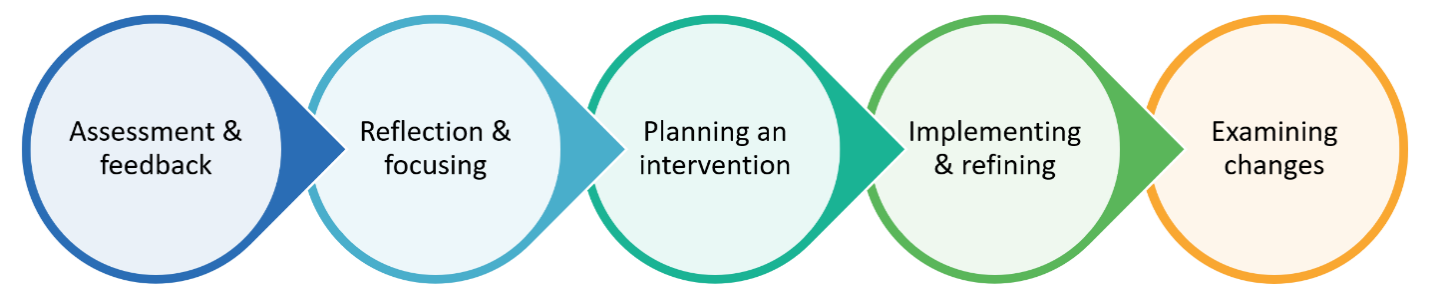
Summary of NSI’s Five-Step Process

The co-researcher (and second author) is a seasoned teacher, having taught a range of subjects for 33 years. At the time of this study, she had already used the CCQ over four consecutive years to improve her teaching practice. The teacher used the Five-Step Process to guide the changes, which has been used extensively in past research efforts with much success (see, for example, Aldridge et al., 2012; Bell and Aldridge, 2014b; Fraser & Aldridge, 2018; Aldridge et al., 2021). Figure 1 provides an outline of the process, and the sections below describe what she did at each step.

### Step one: Collecting the data

The first step of the five-step process is the collection of data using the CCQ. Although many teachers select one class, the co-author elected to administer the CCQ to students in two of her Italian language classes—one year eight and one year 10 class. She explained that “it is interesting to see whether the strategies I am using have similar impacts for students in different classes”. The co-author decided to administer the CCQ in the second half of the year to gauge whether the strategies, which were similar for both classes, provided a suitable learning environment.

### Step two—Reflection and focus

**Fig. 2:**
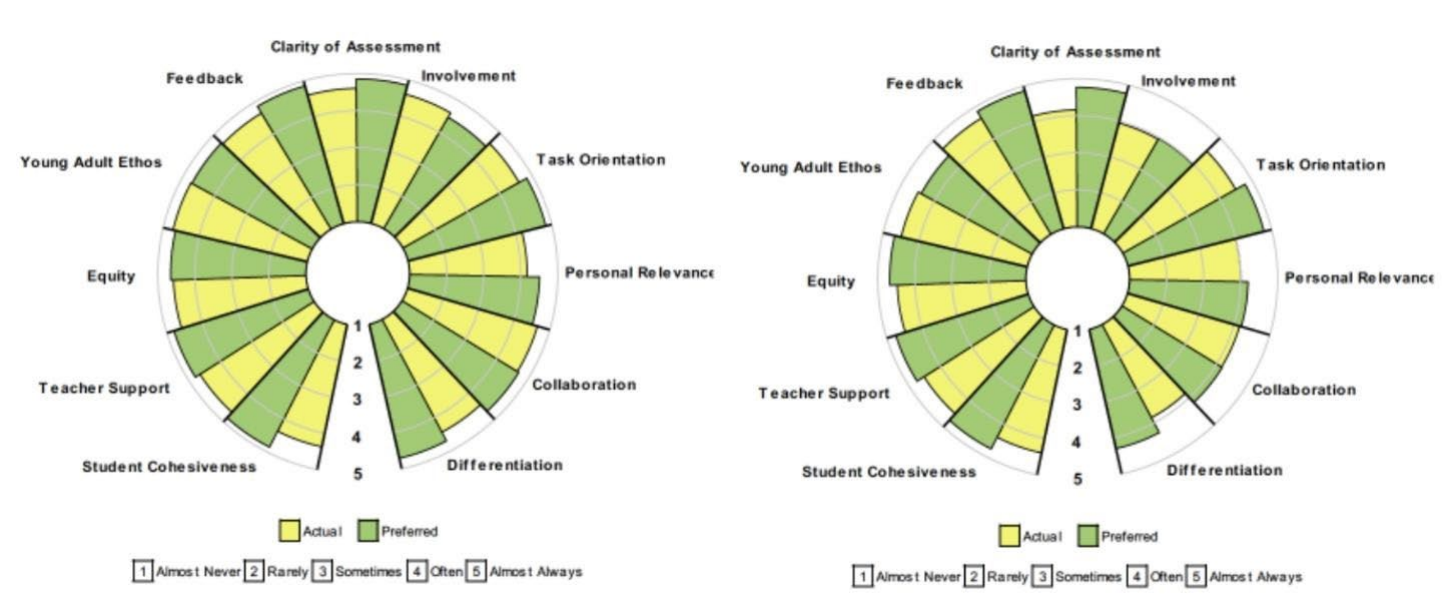
Profiles for the mean scores of the CCQ for students in the teacher’s year 8 (left) and year 10 (right) Italian classes

The second step involved reflecting deeply on the feedback provided by students. Figure 2 provides a graphic portrayal of the year 8 and year 10 students’ responses (average item mean) to the dimensions of the CCQ.

The results suggested that, for both classes, students’ responses to the CCQ were positive, particularly for the relationship dimensions (student cohesiveness, teacher support, and equity). Of this, the teacher noted that “fundamentally, the relationships need to be right. They are at the core of a positive learning environment”. She explained how, for her, relationships involved teachers being themselves. Of this, she said, “Speak to the students as you would to others in your life”. For the teacher, this meant talking to the student with respect and, while maintaining control was important, it was not about being controlling. For her, establishing positive relationships in her class was about being positive about what students were doing, caring about and understanding what they were going through, and “speaking to them as you would another human being.”

The teacher also noted that, despite using the same practices in both classes, the students in year 10 perceived the learning environment less favourably than their counterparts in year 8. For this reason, the findings reported below focused on the year 10 class. For three of the dimensions (involvement, personal relevance, and collaboration), the teacher noted that, even though the students in year 10 scored lower when compared to the year eight students, there was very little difference between the actual and preferred responses. This finding indicated that, despite reporting less of each dimension, they were generally satisfied with them.

The dimensions for which the teacher was most concerned was the clarity of assessment and differentiation. Not only did students in year ten score lower for this dimension, but e actual-preferred discrepancy was greater—suggesting that students would prefer more clarity of assessment. Further, the scale that rated the lowest was differentiation, meaning that students perceived this as happening the least in their classroom.

Once she had finished deliberating, the teacher showed the data to the students. The vignette below describes how the conversation in the teacher’s year 10 class played out.

Although the teacher felt that her year ten students were glad that their voices had been heard, she noted that they were sensitive about the ‘dips’ or lower scores, saying things like “but we love this class”. Her first step was to assure the students that the feedback was not about her but her teaching. She explained that the dips were constructive and that the information was essential to improving what was she was doing in the class. The teacher clarified the importance of critically reflecting on what was she was doing as a means of improvement.

Once the students were comfortable, the teacher continued the discussion. The discussions with each class commenced with what she perceived to be an area to focus. She then opened the conversations to the students, who became engaged and listened as they explained their differing views about the responses to the surveys. Ensuring that the discussion always went back to the data helped the teacher keep the discussion focused and helped determine what areas would benefit from an intervention in the students’ opinion. According to the teacher, there were cases where students agreed with each other, but other instances in which they disagreed. This discussion provided more clarity about what the data reflected in each case. For example, during one of the conversations, a student raised her hand and explained that she disliked grouped work and that she learned better on her own because she liked to be in control of her assessment. After thanking the student for her honesty and sharing, the teacher explained that this information would help improve the classroom for everyone. At this point, others expressed that they disliked the student’s idea and pleaded that group work continues.

The different opinions that students held for preferences in the learning environment highlighted the dilemma faced by teachers in creating a learning environment that suits all students. The teacher sought input on how she might resolve the problem, and the students asked whether they could have a choice. The teacher encouraged them to consider what alternatives might help meet their needs. Ideas included choices about whether they worked together or not and about the choice of topic. At this point, the teacher put forward other ideas, including “a choice of the assessment task, word count, choice of time for an oral presentation”. The teacher said these ideas were a natural presentation that “simply came to me while engaging with the class”. The students reacted positively, engaging with the concept and asking questions about what this would look like for them.

The teacher described the conversation as ‘extensive’; allowing students to think about and talk about their learning and what they needed to improve it. As a result of her reflection and the extended discussions with students, she decided how she could enhance students’ perceptions of two constructs (clarity of assessment criteria and differentiation) by giving the students greater autonomy by involving them in designing their own ‘open’ task.

### Step three: Planning the intervention

The third step involved planning the intervention. As mentioned, the teacher had been using the CCQ for several years, and she had gradually increased the degree of co-construction in the classroom. When referring to the data, she stated:

I decided to undertake co-construction in the form of an open task. Students would be provided with the varying task types, and they would choose the topic and the type of assessment they wanted; … allowing students to learn what they wanted, choosing the length and task type.

The teacher aimed to allow the students to design all aspects of the task and the assessment. For the teacher, the planning involved two steps. The initial planning involved considerations about what topics students might want to be involved in. The second was the consideration of the learning students needed to plan and fulfill their tasks.

**Fig. 3:**
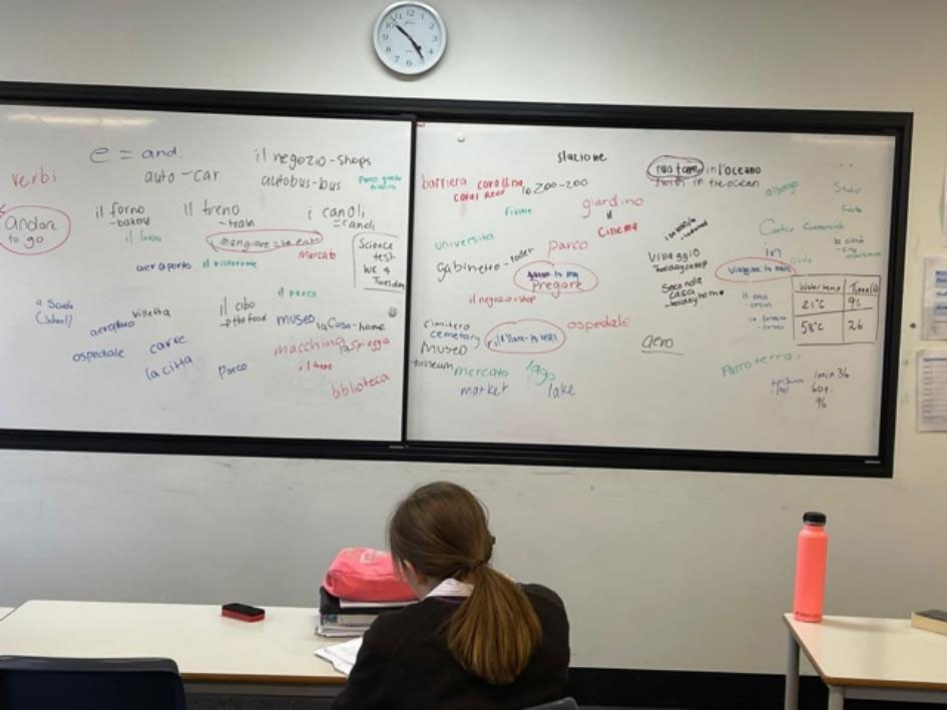
The culmination of the brainstorming activity reported on the whiteboard

The initial planning of the intervention was made quite ‘loose’ to allow students to feel comfortable about offering ideas on topics and providing space for them to be heard and listened to. The planning started with making decisions about the topics that students might choose. Throughout this initial introduction, the teacher made a point of remaining positive and welcoming of the many ideas students thought of. The conversation culminated in a brainstorming session used to share all of the ideas they had introduced during the discussion. The students could come to the board and add ideas that they came up with. Figure 3 provides a picture of the results from the session. Throughout this session, the teacher made a point of remaining positive and welcoming of all student ideas.

Once the teacher had recorded the ideas on the whiteboard, she asked students to decide whether they would prefer to work on a topic of their choice or work on the same topic as other students in the class. Her year ten students opted to work on a topic of their choice.

In preparation for the next step, task creation, the teacher examined the different assessment types that she had planned to use throughout the year and replaced each one with a choice. The teacher decided that students would need to know what assessment tasks would be acceptable and the criteria for success to fulfill the task successfully.

### Step four: Implementing the plan

The fourth step involved implementing the plan. Students came into this step knowing their choice of topic and whether they would work in groups or individually. To help students to make informed decisions about their task creation, the teacher provided information about the assessment tasks available to them and her expectations based on a rubric.

First, the teacher explained to students that, to improve the clarity of the assessment criteria, students must consider what the assessment is or does for each assessment type. For example, she explained what was required for an oral task, conversation, text production, or text analysis. Explaining the tasks took time, and the students asked many questions. According to the teacher, the students were very interested in understanding the information, and they wanted to be clear about their choices. The teacher was clear about the following points concerning the assessment.

- The topics were open and not restricted.
- The tasks did not have specific word counts or time restrictions (for media presentations).
- The teacher decided not to provide examples of assessment tasks as this would stagnate creativity.

Alongside the discussion about the different types of tasks was the need to spend time with the students to help them to understand the rubric. The teacher used the original rubrics that she had drawn up over time based on different assessment types she had offered in the past. She planned each task in detail and included her expectations and how each task would look. Details about the tasks were used to complete the rubric. To help the students, she removed the obstacles, replacing each with a choice (blank) that the students could complete. The students then selected the elements from the rubric that they would assess. During the process, the students asked questions about what different rubric components would look like to have a clearer picture of what they needed to do. Development of the task involved the students selecting elements from within the rubric that they would address.

Once students were clear about the tasks available to them and how they could use the rubric to decide on the elements they would like to assess, the next step involved engaging the student in designing the task. Central to the success of the task was the need for a suitable question. Students began constructing questions that could be of interest in groups. According to the teacher, an important element of this stage was helping students understand what constitutes a good question (concerning their assessment task). To this end, she spent time unpacking what a suitable question would look like and how it would align with the rubric. Given the importance of this step to the success of the open task, an entire lesson was set aside to do this effectively. The teacher stated:

I explained to the students that they could choose what they wanted to learn, how much they wanted to learn, about what they wanted to learn, and that their assessment task would be up to them to develop. This class took full control, choosing the topic, the grammar, and the assessment. If required, I worked with them as a guide, but they decided upon everything. The student engagement and excitement in class were unlike anything I had experienced before. I began to see how important co-construction and student voice are in a classroom.

Once the questions were constructed, students considered the topic they would focus on. Exemplars were provided to help students to do this.

At this point, the teacher became acutely aware that involving the students in the planning made a difference to the enthusiasm with which they engaged in the task. According to the teacher, their involvement made an immediate difference in their desire to engage in the activities.

Of the 18 students, one struggled to decide on a task. Frustrated with her efforts and feeling outside of her comfort zone, the student asked the teacher to provide one. Rather than submit to the suggestion, the teacher worked through the problem with the student, reassuring her that ‘sometimes hard is good’, that it was okay to feel uncomfortable and that “it’s how we work through it [the discomfort] that matters” (The teacher’s words). Eventually, the student decided on her topic, which was highly relevant and personal to her.

### Step five: Evaluating the effectiveness of the intervention

**Fig. 4:**
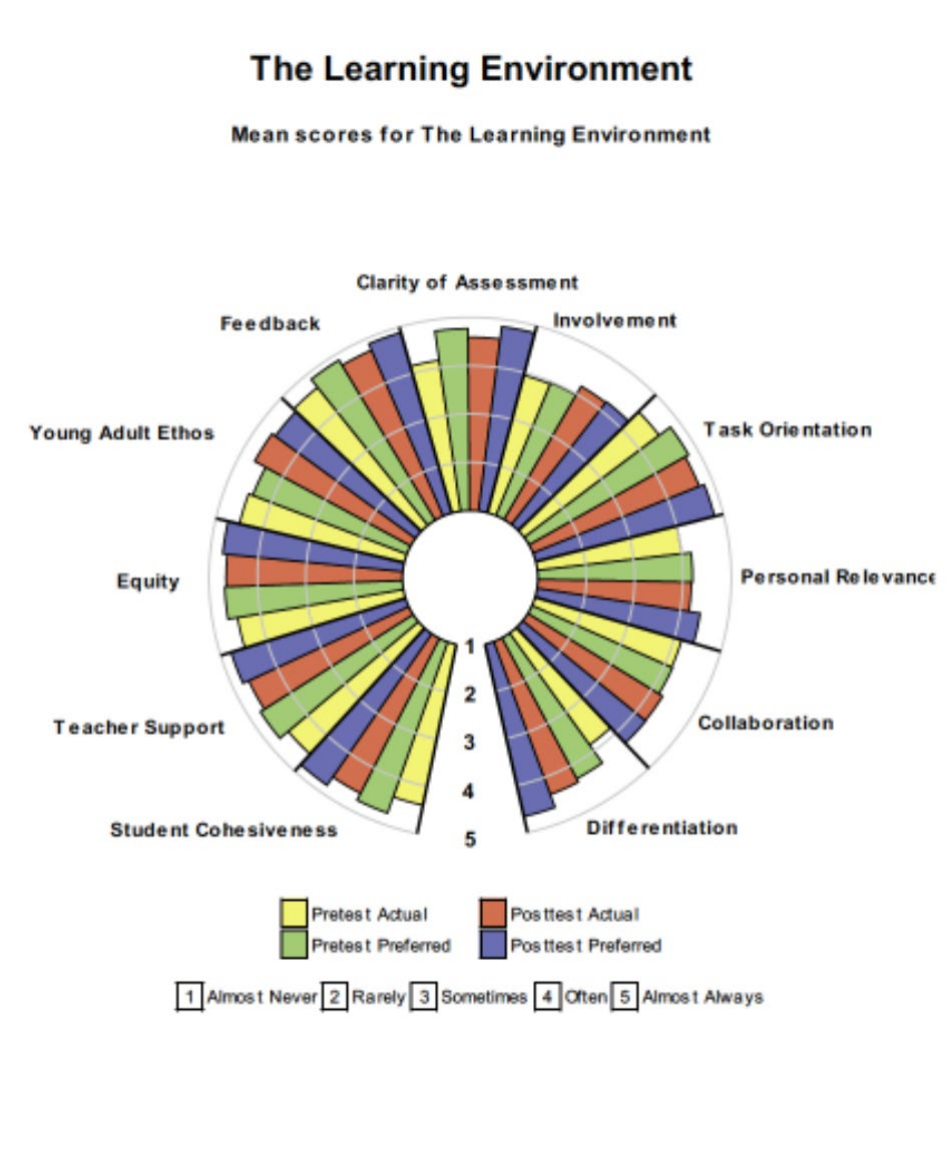
Average item means for the pre-test and post-test scores for year 10 students’ responses to the CCQ

The final step involved evaluating the effectiveness of the intervention. This step involved first, readministering the survey to determine whether there were changes in students’ perceptions of the learning environment and, second, examining students’ narratives. At the end of the intervention period, students responded again to the CCQ. Figure 4 provides a graphic representation of the means scores for the year ten students’ actual and preferred responses to the pre-test and post-test. Bearing in mind that the scores for the pre-test were already high, the changes were affirming. Improvements in the average item mean were reported for all dimensions of the CCQ (see Fig. 4; Table 2). Further, the standard deviation for the post-test, reported in Table 2, was lower for the post-test, indicating that the spread of scores was narrower when compared to the pre-test results. Given that the learning environment dimensions are interrelated, it is not unusual for one dimension to affect other dimensions.

**Table 2:** Means, standard deviations and differences for pre-test and post-test scores on the CCQ—Year 10 students

Scale	Mean		Std. Deviation		Difference	
	Pre-test	Post-test	Pre-test	Post-test	Effect size	t
Student Cohesiveness	4.40	4.60	0.41	0.38	0.51	1.50
Teacher Support	4.52	4.65	0.46	0.38	0.31	0.88
Equity	4.48	4.65	0.51	0.35	0.39	1.15
Responsibility for Learning	4.50	4.67	0.41	0.28	0.48	1.43
Teacher Feedback	4.66	4.74	0.42	0.39	0.12	0.63
Clarity of Assessment Criteria	4.16	4.59	0.44	0.41	1.15	3.03**
Involvement	3.94	4.19	0.83	0.59	0.35	1.01
Task Orientation	4.50	4.71	0.46	0.34	0.52	1.52
Personal Relevance	4.00	4.18	0.73	0.66	0.26	0.75
Collaboration	4.16	4.27	0.61	0.58	0.18	0.57
Differentiation	3.84	4.38	0.79	0.59	0.77	2.27*

*N* = 17 students who were present for both the pre-test and the post-test

The pre-post differences were statistically significant for two constructs: assessment criteria (effect size > 1.15) and differentiation (effect size 0.77). The effect sizes for these two constructs are considered large according to Cohen (1977), and therefore of educational importance. As one of the students stated in her narrative: “The idea of directing our learning and studying a subject designed with the flexibility to support how we learn best was a turning point for us.”

For The teacher, these results were affirming. She stated that using the open task “heightened [students’] engagement and commitment to the task. The ownership of their learning increased their purpose for learning, which impacted the result of the CCQ and their tasks”.

Except for the student who struggled initially, as noted at Step 3, they generally agreed that choosing their topic was beneficial. One student wrote: “I worked harder to discover more about my chosen topic and ask more questions because my passion heightened my curiosity to learn”. Another student wrote, “The idea of directing our learning and studying a subject designed with the flexibility to support how we learn best was the turning point for us”.

In implementing the open tasks, students were made aware of co-construction and student voice. Of the process, one student wrote, “co-construction [provides] a process that allows me to create my own tasks and assessments so that my learning can relate to me”. The student went on to describe that, “In class, we are allowed to choose how to present what we learned. Everyone in my class had different ideas as to how they were going to present.”

At the end of the process, students considered how co-construction may have impacted their learning. There was a range of responses, with some students identifying the benefits of being more interested in the subject. One student wrote:

We have had every opportunity to voice our suggestions and concerns regarding our learning. Having a teacher who understands the importance of student voice and is willing and able to accommodate each individual’s wants and needs has been key to our success.

Others considered how the process had provided them with a deeper understanding of the subject and two students expressed how co-construction had improved their learning. One of the students wrote,

As a result of co-construction, I have improved my learning by analysing and evaluating how I choose to learn. I am also able to recognise strategies that assist with understanding the requirements of a task. These skills are transferable to other subjects and education endeavours beyond school, as it stimulates the idea of self-directed learning.

For he teacher, the introduction of co-construction in her teaching has been ongoing since she first embarked on using the CCQ in 2016. Of this, she stated, “The original introduction to the process has now impacted and changed every lesson that I teach. I am no longer restricted or limited by the syllabus, and I adapt the syllabus where possible to the wants of the students, involving them in planning for the year. This has been an ongoing adjustment to my teaching, particularly in the senior classes from this experience”.

When asked how she felt about using the CCQ, she responded, “I can see why some people might be concerned—uncomfortable—about getting student feedback [using the CCQ], but for me, this is about an opportunity to change or improve what I do. I can’t see a negative. This is a journey, a chance to examine what the people that I engage with the most have to say [about my teaching and interactions with them].” The teacher went on to explain how her journey, involving co-construction, has allowed her to hone her skills and, in her own words, “relinquished [her] usual teaching practice and given over to complete student voice”.

According to the teacher, Despite the apparent benefits of co-construction, it was not without challenges. One of the biggest challenges, according to the teacher, was the amount of time outside of lessons that were spent supporting students in various ways. In the teacher’s opinion, being accessible to the students was necessary, and she felt that students might have floundered or lost momentum without it. This support was often provided outside of classroom time slots (through emails and meetings), demanding time and energy.

For the students, the shift to co-construction also presented challenges. One of the students explained that the task determined in co-construction required skills such as time management and the need to be self-directed in their learning—which some students may have been less prepared for. Having said this, the same student indicated that she had learned a range of skills that would be transferable to other subjects.

## Discussion

This study has, like all studies, some limitations. First, the sample was restricted to one teacher and her class. Given our case study approach, focusing on one teacher provided a detailed account of the journey. However, generalisation to other teachers should be made with caution. Second, the study was carried out in an all-girls school; therefore, generalising the results to other settings should also be done with this in mind. Given these limitations, we recommend that future studies involve teachers with different experience levels and start points (e.g., concerning the pre-test) to examine the efficacy of using a learning environment instrument as a starting point to co-construction.

Despite these limitations, our findings suggest that using the CCQ as part of a reflective process effectively assisted co-construction. The introduction of open tasks led to a statistically significant (*p* > 0.05) improvement in students’ perception for two of the CCQ scales (clarity of assessment criteria and differentiation). It would appear that giving students a sound understanding of marking rubrics and the opportunity to design their assessment led to a more positive perception of the clarity of assessment criteria scale. In addition, there was a statistically significant improvement for the differentiation dimension. This finding indicates that the use of open tasks allowed for individualisation of the learning for students.

The teacher reflected on her teaching practice, initially using the CCQ and then throughout the process. This critical self-reflection allowed her to consider the students’ needs and change her teaching practice to accommodate these needs. Notably, the teacher also encouraged students to reflect on their learning as part of the process. For the students, the activities provided by the teacher and the discussions with the teacher and their peers encouraged reflection on learning, helping them develop an active understanding of their learning (Watkins et al., 2002).

Central to the use of the CCQ feedback was the teacher’s dialogue with the students. These discussions took into consideration the perspectives of all students and allowed the teacher to explore the experiences and opinions of individuals. In her discussions with students, the teacher encouraged openness and, even though some of them were not in agreement, she encouraged others to listen to these opinions. In this way, the teacher heard the full range of perspectives, allowing her to create a learning environment that better suited a range of students’ needs.

Based on her reflection on the pre-test data and discussion with students, The teacher decided to provide students with open tasks. Often teachers feel constrained by the externally mandated curriculum and the need to cover the content. However, the teacher’s decision to offer an open task overcame this restraint and was necessary from a critical theory perspective. This decision embraced and empowered students’ critical voice to help her implement change, making co-construction in her classroom a reality.

Students’ perceptions of the relationships dimension, particularly the teacher support scale, were high in the pre-test. This high score is notable given that one of the keys to successful co-construction is building strong, positive, trusting relationships between students and between the teacher and the students. These relationships allow the teacher to deeply know and understand the students as individuals, including their perspectives, passions, likes, and social networks. Fostering deep and sincere relationships helps teachers better understand their students and create a shared understanding of what is important in the classroom setting. Her already strong relationship with the students brings into questions whether the introduction of open tasks would have been successful in a learning environment with less positive relationships.

The teacher’s decision to provide students with an open task is likely to give students a better understanding of their learning and a greater propensity to take responsibility for their learning. Given that young people enjoy and learn well when they have agency and choice over what they do (Lodge), they are likely to learn skills that help them self-regulate (plan, monitor, evaluate).

## Conclusions

Co-construction holds much promise for providing students with agency over what and how they learn. Achieving co-construction requires that teachers have a deep understanding of their student’s learning needs. The Classroom Climate Questionnaire (CCQ) provides information from the students’ perspective that teachers can use to understand the students’ needs better and critically reflect on their practice. Feedback from the CCQ can also be used as a springboard for generating discussions in the classroom that will better enable the co-construction of the learning environment.
